# A Case of Weight Gain and Edema With Difficulty in Moving Legs Due to Intravascular Large B-cell Lymphoma Diagnosed by Skin Biopsy

**DOI:** 10.7759/cureus.51051

**Published:** 2023-12-24

**Authors:** Shintaro Imaoka, Masaya Maegaki, Daisuke Son, Toshihiro Hamada, Shin-ichi Taniguchi

**Affiliations:** 1 Department of Community-Based Family Medicine, Tottori University, Yonago, JPN; 2 Department of Hematology, Tottori University, Yonago, JPN

**Keywords:** weight gain, lactate dehydrogenase, intravascular large b-cell lymphoma, difficulty in moving legs, edema

## Abstract

We report a case of intravascular large B-cell lymphoma (IVL) with spinal cord involvement. A 76-year-old woman was referred to our department due to generalized edema and weight gain. She also had difficulty moving her legs. She had no superficial lymphadenopathy upon examination. Her laboratory tests showed a markedly elevated blood lactate dehydrogenase (LDH) level. Although heart failure or interstitial lung disease was initially suspected, she was diagnosed with IVL by skin biopsy. An MRI revealed spinal cord involvement. Post-hospitalization, she began rituximab-combined chemotherapy. In this case, we considered that the spinal cord involvement of the lymphoma caused the neurogenic bladder and leg weakness. IVL often infiltrates the central nervous system and presents with neurological symptoms, including neurogenic bladder. Therefore, imaging studies should be planned to search for the involvement of the central nervous system in lymphoma if accompanied by neurological symptoms. In addition, in patients with a markedly elevated LDH or soluble interleukin-2 receptor level without lymphadenopathy, IVL should be suspected, and consultation with hematologists should be considered.

## Introduction

Intravascular large B-cell lymphoma (IVL) is a malignancy that mainly involves the blood vessels. Its clinical symptoms vary, and its diagnosis is often difficult due to nonspecific clinical findings. The clinical course of IVL is rapid and progressive; in many cases, the diagnosis is made only during autopsy because it is often impossible to diagnose the disease before death [1]. If a patient is diagnosed before death, rituximab-combined chemotherapy can improve the prognosis [2]. Herein, we report a case of IVL in a patient who presented with weight gain and generalized edema, which was diagnosed by skin biopsy.

## Case presentation

A 76-year-old woman was referred to our department due to generalized edema and weight gain. She has had dry eyes and hyposalivation since 74 years of age. Starting three weeks before referral, she noticed edema on both lower legs, face, and hands, and she lost her appetite. The edema on both lower legs progressed until her legs felt heavy when she walked. She also began to have difficulty moving her legs when getting into the bathtub, eventually requiring assistance from her family. She also had difficulty urinating, and her weight increased from 51.5 kg to 56 kg within two weeks.

At the first visit, she was 150 cm tall and weighed 56 kg. Her body temperature was 36.3℃, with the other vital signs being normal. Chest auscultation revealed clear breath sounds and no heart murmur. The liver and spleen were nonpalpable. There was no superficial lymphadenopathy in the neck, axilla, or inguinal region, and no abnormal rash on the trunk. There was mild edema of the face and back of both hands, but an obvious nonpitting edema of both lower legs was noted. The patient could stand, hold a standing position, and walk slowly but independently. Blood tests showed elevated C-reactive protein and lactate dehydrogenase (LDH) levels (Table 1).

**Table 1:** Laboratory data on the first visit and five days after the first visit MCV: mean corpuscular volume, RDW: red cell distribution width, AST: aspartate aminotransferase, ALT: alanine aminotransferase, γ-GTP: γ-glutamyl transpeptidase, CRP: C-reactive protein, BUN: blood urea nitrogen, LDH: lactate dehydrogenase, BNP: brain natriuretic peptide, IL-2R: interleukin-2 receptor

Test	Level	Reference Range
Peripheral blood		
White blood cells (/μL)	8900	3600-8900
Neutrophils (%)	86	38-74
Lymphocytes (%)	4	16-49
Monocytes (%)	9	2-10
Eosinophils (%)	1	0-3
Basophils (%)	0	0-8
Red blood cells (×10^6^/μL)	3.78	3.8-5.0
Hemoglobin (g/dL)	10.7	11.5-15.0
Hematocrit (%)	32.6	35-44
MCV (fL)	86.2	83-96
RDW (%)	14.9	12-15
Platelets (×10^4^/μL)	15	14.5-34.0
Blood chemistry		
Total protein (mg/dL)	5.6	6.5-8.3
Albumin (mg/dL)	2.5	3.8-5.3
AST (U/L)	39	8-38
ALT (U/L)	31	4-43
γGTP (U/L)	69	<48
CRP (mg/dL)	6.07	<0.30
BUN (mg/dL)	12.5	8-20
Creatinine (mg/dL)	0.66	0.40-1.10
Na (mEq/L)	131	135-150
K (mEq/L)	4.4	3.5-5.3
Cl (mEq/L)	97	98-110
Ca (mg/dL)	7.9	8.8-10.2
LDH (U/L)	1276	121-245
BNP (pg/mL)	27.6	<18.4
Additional tests 5 days after the first visit		
Total bilirubin (mg/dL)	0.5	0.2-1.2
SIL-2R (U/mL)	4317	122-496

**Figure 1:**
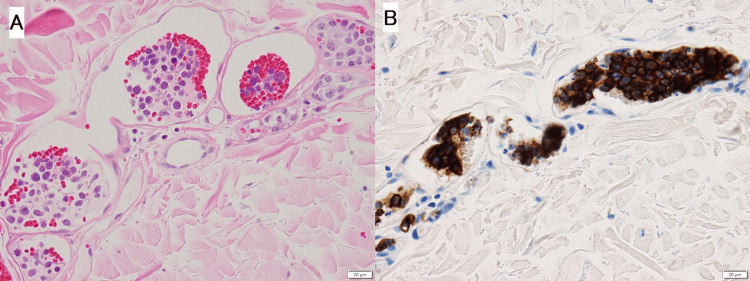
Histopathological images of the skin biopsy (A) Skin biopsy showing capillary lumina filled with large abnormal lymphocytes with large nuclei (HE staining ×40). (B) Large abnormal lymphocytes show positive for CD20 (CD20 staining ×40)

A plain CT of the chest and abdomen showed mild hepatomegaly but no lymphadenopathy. No evidence of heart failure was noted by echocardiography. When she was referred to the hematology department five days after her first visit, she had a high soluble interleukin-2 receptor (SIL-2R) level. Bone marrow and skin biopsies were planned to determine the presence of a lymphoma. The bone marrow biopsy showed no tumor cells. Meanwhile, random skin biopsies were performed at five sites from the abdomen’s left side to the lower abdomen and both thighs. The samples from four sites showed proliferation of large abnormal lymphocytes in the dermal to subcutaneous vasculature that were positive for CD20 (Figure 1), CD5, and CD79a and negative for CD3 by immunostaining. No extravascular proliferation of tumor cells was observed. Based on these results, she was diagnosed with IVL.

**Figure 2:**
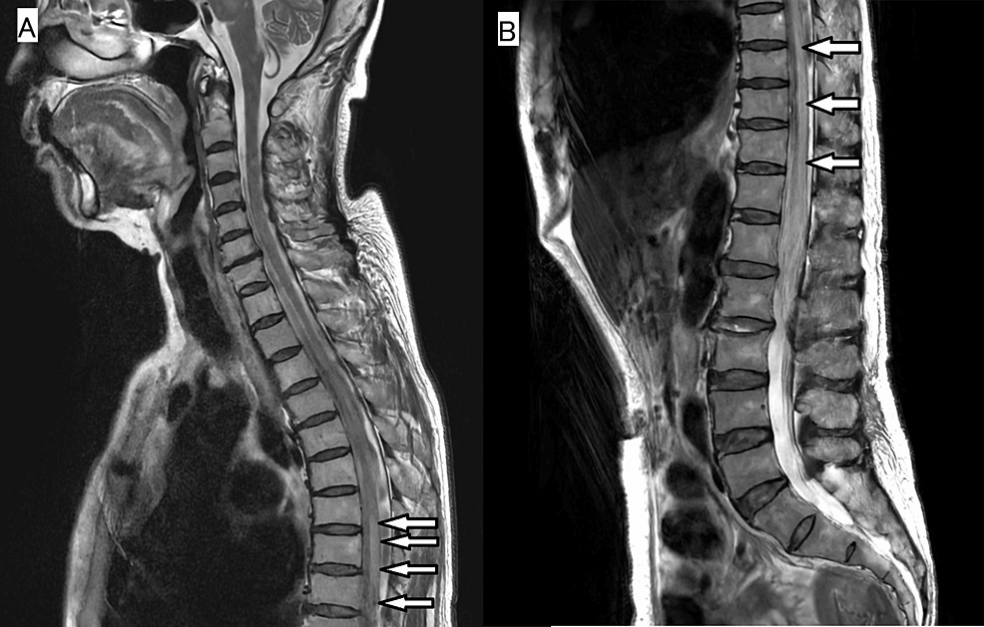
MRI of the spinal cord T2-weighted image shows an intramedullary lesion with high signal intensity from the Th7 level (A) to the spinal cord cone (B)

Twenty-five days after her first visit, she was hospitalized due to increased weakness in both lower legs. A plain MRI of the spinal cord showed a series of T2 high-signal areas centered in the gray matter from the Th7 level to the spinal cord cone (Figure 2), suggesting the presence of spinal cord involvement in the lymphoma. Post-hospitalization, she began treatment with R-CHOP (rituximab, cyclophosphamide, doxorubicin, vincristine, and prednisone).

## Discussion

We encountered a patient who had difficulty moving both legs due to spinal cord involvement in the IVL. IVL is characterized by the intravascular proliferation of lymphoma cells. Its diagnosis is often difficult because of the absence of lymphadenopathy in the case. Recently, more cases of IVL have been diagnosed by random skin biopsy, which is recommended when IVL is suspected, regardless of the presence or absence of a skin rash [3]. In our case, the patient was referred to the hematology department to investigate the cause of the elevated LDH levels, resulting in an IVL diagnosis by skin biopsy. Our patient did not have a fever. In a Japanese cohort study of IVL, fever was present in 78% of IVL patients [2]. Therefore, we considered that the absence of fever in this case was atypical for IVL. At the time of her first visit, we initially considered heart failure because of the edema on both of her lower legs and weight gain. Additionally, we also considered interstitial lung disease associated with Sjögren’s syndrome (SS) because, two years earlier, she underwent a thorough evaluation for dry eyes and hyposalivation. Schirmer's and Gumm’s tests were positive, but she was negative for anti-SS-A/B antibodies. Although a definitive diagnosis of SS was not made at that time, we thought that the interstitial lung disease associated with SS might have developed during the interim; however, chest CT and echocardiography showed no significant abnormal findings suggestive of heart failure or interstitial lung disease. Meanwhile, her blood LDH level was >1,000 U/L, indicating the possibility of a malignant tumor. The chest and abdominal plain CT results showed no obvious neoplastic lesions, hence the referral to the hematology department for further evaluation for leukemia and lymphoma, leading to bone marrow and skin biopsies. The SIL-2R level was also elevated in this case. The SIL-2R levels are also elevated in many IVL cases, with up to 70% of cases having SIL-2R levels of >5,000 U/mL [4]. Given that liver disease was also a differential disease in cases with elevated LDH levels, the patient was referred to our hospital’s hepatology department, and liver tissue examination was considered if no other cause could be identified; however, liver biopsy was not performed because the diagnosis of IVL determined by skin biopsy was sufficient to explain this clinical course.

The suspected cause of the patient’s difficulty in walking widely varied from that during the initial consultation to that after the confirmation of the diagnosis. During her first visit, marked edema of the legs was considered the main cause of difficulty in moving both lower legs, and a spinal cord abnormality was not suspected. Additionally, the dysuria during her first visit was caused by a neurogenic bladder secondary to spinal cord involvement of the lymphoma. Notably, IVL often presents with neurological symptoms [5]. If we had associated the tumor with bilateral lower leg weakness and a neurogenic bladder, a spinal abnormality could have been suspected earlier. Although IVL was diagnosed while the patient was still alive, it took approximately one month to confirm the diagnosis, during which time the patient’s ability to perform activities of daily living decreased and the burden of caring for the family increased. It was a point of reflection for us.

## Conclusions

Patients with IVL present with a variety of clinical symptoms that are likely encountered by internal medicine physicians. IVL often infiltrates the central nervous system and presents with neurological symptoms, including a neurogenic bladder. Therefore, imaging studies should be planned to search for the involvement of the central nervous system in lymphoma if accompanied by neurologic symptoms. Given that IVL is a rapidly progressive and potentially fatal disease, it is important to include it as a differential diagnosis in cases with elevated LDH levels. Additionally, SIL-2R levels are also elevated in many IVL cases. Therefore, in patients with elevated LDH and SIL-2R levels, a consultation with a hematologist is recommended to evaluate for the presence of IVL even in the absence of lymphadenopathy.
